# Oxytocin and orexin systems bidirectionally regulate the ability of opioid cues to bias reward seeking

**DOI:** 10.1038/s41398-022-02161-z

**Published:** 2022-10-04

**Authors:** Giuseppe Giannotti, Francesca Mottarlini, Jasper A. Heinsbroek, Mitchel R. Mandel, Morgan H. James, Jamie Peters

**Affiliations:** 1 Department of Anesthesiology, University of Colorado, Anschutz Medical Campus, Aurora, CO 80045 USA; 2 Department of Pharmacological and Biomolecular Sciences, University of Milan, 20133 Milan, Italy; 3 Department of Psychiatry, Robert Wood Johnson Medical School, Rutgers University, Piscataway, NJ 08854 USA; 4 Department of Pharmacology, University of Colorado, Anschutz Medical Campus, Aurora, CO 80045 USA

**Keywords:** Addiction, Neuroscience

## Abstract

As opioid-related fatalities continue to rise, the need for novel opioid use disorder (OUD) treatments could not be more urgent. Two separate hypothalamic neuropeptide systems have shown promise in preclinical OUD models. The oxytocin system, originating in the paraventricular nucleus (PVN), may protect against OUD severity. By contrast, the orexin system, originating in the lateral hypothalamus (LH), may exacerbate OUD severity. Thus, activating the oxytocin system or inhibiting the orexin system are potential therapeutic strategies. The specific role of these systems with regard to specific OUD outcomes, however, is not fully understood. Here, we probed the therapeutic efficacy of pharmacological interventions targeting the orexin or oxytocin system on two distinct metrics of OUD severity in rats—heroin choice (versus choice for natural reward, i.e., food) and cued reward seeking. Using a preclinical model that generates approximately equal choice between heroin and food reward, we examined the impact of exogenously administered oxytocin, an oxytocin receptor antagonist (L-368,899), and a dual orexin receptor antagonist (DORA-12) on opioid choice. Whereas these agents did not alter heroin choice when rewards (heroin and food) were available, oxytocin and DORA-12 each significantly reduced heroin seeking in the presence of competing reward cues when no rewards were available. In addition, the number of LH orexin neurons and PVN oxytocin neurons correlated with specific behavioral economic variables indicative of heroin versus food motivation. These data identify a novel bidirectional role of the oxytocin and orexin systems in the ability of opioid-related cues to bias reward seeking.

## Introduction

Drug choice and the ability of drug cues to motivate drug seeking are conceptually dissociable processes that contribute to the pervasive opioid taking and seeking that characterize opioid use disorder (OUD). Over the past several decades, preclinical models of relapse have been the focus of efforts to advance OUD therapies. In contrast, preclinical models of opioid choice have only recently begun to receive significant attention. The vast majority of these preclinical choice models have revealed that rodents will primarily choose natural rewards (e.g., food, social reward) over drug rewards under conditions of mutually exclusive choice, equivalent price (e.g., lever-press requirements for reward), and equal delay [1]. Thus, some have suggested these models capture components of contingency management therapy, as they typically lead to voluntary abstinence and reduced relapse rates for opioids [2–4]. Unfortunately, contingency management has a relatively low success rate for treating OUD long-term, and not all individuals with OUD respond to it [5]. Thus, models that identify subpopulations of opioid-choosers are vital for screening novel potential pharmacotherapeutics for OUD. We recently developed a preclinical choice model that identifies a subpopulation of rats that choose heroin over food and yields increased reward seeking for heroin cues over food cues [6]. Thus, we are uniquely positioned to screen novel potential OUD therapies that target choice behavior to reduce opioid taking and seeking.

Oxytocin is a neuropeptide produced by a subset of neurons located in the paraventricular nucleus of the hypothalamus (PVN). Aside from its known role in parturition and maternal behaviors [7], oxytocin plays an important role in social reward processes [8–10], anxiety [11, 12] as well as stress responsivity [13, 14]. Moreover, increasing evidence indicates that oxytocin has anti-addictive properties, pointing to this neuropeptide as potential treatment for multiple abused substances [15, 16]. In rats, systemic administration of oxytocin decreases the motivation to self-administer methamphetamine [17], cocaine [18], and alcohol [19, 20], and oxytocin decreases cue-induced methamphetamine [17, 21] and cocaine [18] seeking, as well as stress-induced alcohol seeking [22, 23]. In humans, intranasal oxytocin treatment reduces alcohol withdrawal symptoms [24], craving [25], and metabolic brain activity triggered by alcohol-associated cues [26], and it reduces craving scores in patients with heroin [27] and cocaine [28] use disorders. Together these data underscore the potential efficacy of oxytocin-based therapies to reduce relapse for multiple abused drugs [29].

Orexins A and B are neuropeptides produced in the lateral hypothalamus (LH) that regulate motivated drug seeking. Orexin-expressing neurons are activated by stimuli associated with morphine, cocaine, and alcohol [30–35], and the magnitude of their activity is directly related to drug seeking behavior [36, 37]. The overwhelming majority of studies have focused on the orexin receptor 1 (Ox1R) as the primary site of orexin signaling in drug reward [38]. For example, systemic treatment with the Ox1R receptor antagonist SB-334867 decreases motivated responding and cue-induced reward seeking for all drugs of abuse tested, including the opioids oxycodone, heroin, fentanyl, and remifentanil [30, 39–41]. Although the role of orexin receptor 2 (Ox2R) signaling has been studied less extensively in drug reward, systemic or central administration of Ox2R antagonists reduces heroin and alcohol self-administration at doses that do not impact food self-administration [42, 43]. These data raise the possibility that compounds that block signaling at both the Ox1R and Ox2R (dual orexin receptor antagonists; DORAs) might have more pronounced therapeutic properties compared to single orexin receptor antagonists [44–46]. Indeed, initial preclinical studies indicate that DORAs reduce drug taking and seeking across several classes of drugs of abuse [47–50], and a preliminary clinical study reported that the DORA suvorexant (marketed by Merck as Belsomra^TM^) reduces several relapse-related and self-reported craving indices in patients with cocaine or opioid use disorder [51, 52]. Based on the success of these studies, the National Institute on Drug Abuse (NIDA) declared the orexin system a target of high priority for new medication development to tackle the opioid epidemic [53], although additional studies are needed to examine the efficacy of DORAs in preclinical OUD models.

Despite the growing popularity of choice models in preclinical addiction research, to date no study has investigated the contributions of hypothalamic neuropeptide systems under choice conditions. To address this gap in knowledge, we tested whether pharmacological treatment with either oxytocin or a DORA could alter choice behavior, under conditions when rewards are available or unavailable (i.e., when seeking is driven by reward-related cues). We also applied principles from the field of behavioral economics [54] to investigate the motivation for heroin versus natural reward (food) and assess the relationship between behavioral economics variables and the number of hypothalamic oxytocin and orexin neurons. We found that pharmacological modulation of the orexin and oxytocin systems did not alter heroin choice when rewards were available, but that manipulations of these systems specifically reduced heroin seeking under conditions where both heroin and food cues, but not the rewards, were present. Moreover, the number of oxytocin- and orexin-expressing neurons correlated with specific economic demand variables for each reward. These data support a central role of the hypothalamic system in specific outcome measures related to OUD, and add to a growing body of literature suggesting therapeutic strategies that modulate oxytocin and/or orexin signaling are promising avenues for reducing opioid relapse.

## Materials and methods

### Animals

All animal procedures followed guidelines approved by the University of Colorado Anschutz Medical Campus Institutional Animal Care and Use Committee. Subjects were age-matched (P55–60) male (*n* = 32) and female (*n* = 32) Wistar rats (Charles River, Raleigh, NC). Animals were single-housed in a temperature and humidity-controlled environment (lights on 8am-8pm) with free access to standard laboratory chow (Envigo 2020X) and water. Procedures followed the guidelines outlined in the Guide for the Care and Use of Laboratory Animals [55].

### Experimental agents

Synthetic human oxytocin (Cell Sciences, CRO300GB) was dissolved in sterile water and administered at doses of 1 or 3 mg/kg (1 ml/kg, i.p.) 30 min prior to testing. This pretreatment interval and dose range were based on prior literature showing an effective reduction in drug seeking with minimal side effects [17, 56, 57]. The oxytocin receptor antagonist L-368,899 hydrochloride (Tocris, 2641) was dissolved in sterile water and administered at a dose of 5 mg/kg (1 ml/kg, i.p.) 30 min prior to testing, which has been shown to effectively block the effects of oxytocin in rats [58]. The dual orexin receptor antagonist DORA-12 (Merck) was dissolved in 5% DMSO + 40% captisol in sterile water and administered at doses of 10 mg/kg (1 ml/kg, i.p.) or 30 mg/kg (3 ml/kg, i.p.) based on effective doses of other DORAs reported in the literature [47–50]. Heroin (diamorphine hydrochloride) was dissolved in 0.9% saline (0.04 mg/50 μl infusion per reward). Dustless precision grain-based food pellets (45 mg each, Bio-Serv, F0165) were delivered in 3-pellet quantities per reward.

### Food and heroin self-administration

Rats were surgically implanted with an intravenous jugular catheter as previously described [6]. After recovery, rats were trained to self-administer food and heroin in tandem over daily (weekday) sessions inside standard rat operant chambers (Med Associates, St. Albans, VT, USA). During the first 30 min of each session, the food (right) lever and cue (3.5 kHz tone, 5 sec) were available, followed by a 10 min time-out with levers retracted. Thereafter, the heroin (left) lever and cue (light above lever, 5 sec) were available for 2 h. Training began on a fixed-ratio 1 (FR1) schedule of reinforcement. Cues for each reward were initiated simultaneously with reward delivery onset, and levers retracted during cue presentation. After 8 sessions, training progressed through 8 additional FR steps (FR3, FR8, FR20, FR50, FR125, FR313, FR783, FR1958) with at least 1 day on each FR. To prevent infection and catheter occlusion, respectively, cefazolin and taurolidine citrate solution (TCS) were administered after each self-administration session. Catheter patency was periodically verified using methohexital sodium (1 mg/0.1 ml, i.v.). Five rats were excluded from the final dataset due to defective catheters.

### Behavioral economics and demand curves

**Fig. 1:**
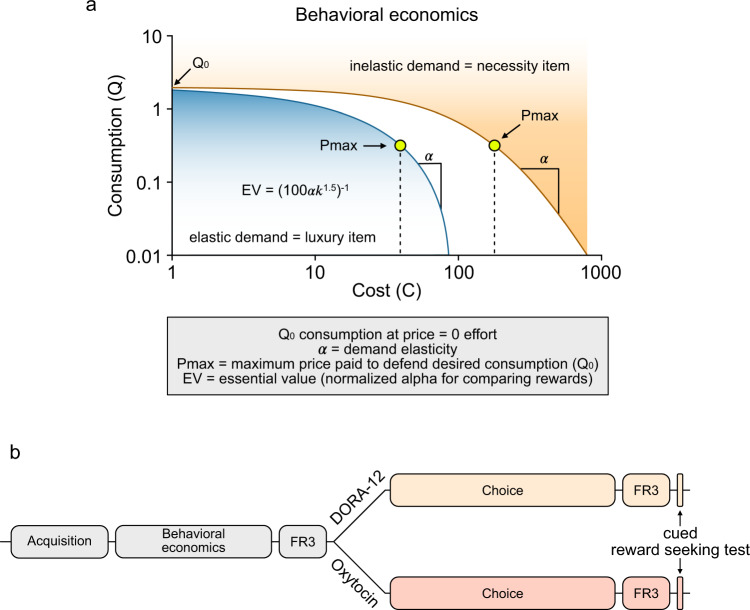
Behavioral economics principles and experimental timeline. **a** Example of “elastic” and “inelastic” demand curves and definition of the behavioral economic parameters analyzed in the present study (gray box). **b** Experimental timeline for heroin and food self-administration, behavioral economic, choice testing and cued relapse. Animals where trained to self-administer food (30 min) and heroin (2 h) in tandem for 8 sessions (FR1), followed by 8 additional fixed-ratio steps used to extract behavioral economics variables. After 3 days of self-administration under FR3 schedule, animals where split into two different treatment groups (DORA-12 and Oxytocin) and entered the choice phase. At the end of choice testing, animals received 3 days of food and heroin self-administration under FR3 schedule before being tested in the cued reward seeking test.

We used a between-session behavioral economics paradigm wherein the price (i.e., FR requirement) for rewards increased over daily sessions [59]. Food and heroin demand curves were generated for each animal, using the responding on each FR price point, according to the formula [54]:

$$\ln Q = {{{\mathrm{ln}}}}Q_0 + k(e - \propto Q_0C - 1)$$

where *C* indicates consumption (i.e., number of food or heroin rewards earned at each FR step) and constant k (set to 8.85) specifies the range of consumption values [60]. An elastic demand curve, one in which consumption drops off rapidly with increasing price, suggests the animal considers the reward a “luxury item.” Whereas an inelastic demand curve, one in which increases in price are well-tolerated to defend desired consumption, suggests the animal considers the reward a “necessity item.” Fig. 1a shows an example of an elastic versus inelastic demand curve, illustrating these concepts. From the resulting demand curves, we extracted the following variables: *α* (demand elasticity; rate at which consumption declines with increasing price), *Q*_0_ (consumption at null cost; intrinsic motivational efficacy of the reward), and *P*_max_ (maximum price paid to defend desired consumption; derived using a solver algorithm) [54, 59, 61]. We also calculated the essential value (EV) of each reward according to the formula:

$${\it{{\rm{EV}}}} = (100\alpha k^{1.5})^{ - 1}$$

Because EV is independent of reinforcer magnitude, it is useful for comparing different reinforcers directly [59, 62]. Therefore, we report both α and EV here, as we want to compare directly between heroin and food rewards. Figure 1a defines each of these behavioral economics metrics of motivational state and illustrates their relationship to the demand curve.

### Choice between food and heroin

Choice procedures were implemented after self-administration training. During choice sessions, rats were given 14 trials to choose food or heroin (FR3). Once the FR requirement was reached on one lever, the respective reward and cue was delivered, then levers retracted for a 10 min time-out between trials. The first 2 choice sessions began with two forced-choice trials for each reinforcer (4 total trials, completed by all rats) during which only the food or heroin lever was extended in alternating order, followed by 10 free-choice trials with both lever options. Thereafter, the forced choice trials were dropped and only free-choice sessions (both lever options for all 14 trials) were conducted. Following at least 6 stable choice sessions (≤20% variability in total choices over the last 2 sessions), rats began repeated choice testing, with each test separated by two choice sessions. Heroin choice was expressed as the percentage of heroin choices over total choice trials. Importantly, there were no omissions; all rats completed all 14 trials on each test. Choice sessions (including choice tests) were the only behavioral sessions where both food and heroin rewards were available simultaneously, with both food and heroin levers extended at the same time. Rats were assigned to the oxytocin or orexin study in a balanced manner based on the percentage of heroin choice and sex. Figure 1b depicts the experimental timeline, and the splitting of treatment groups. Oxytocin and orexin drugs were administered 30 min prior to placement in the behavioral chamber for testing. Oxytocin rats received vehicle (water) or oxytocin (1 or 3 mg/kg, i.p.). Since no effect of oxytocin on opioid choice was observed (see Results), rats underwent an additional test with vehicle (water) or the oxytocin antagonist L-368,899 (5 mg/kg, i.p.) to assess the involvement of the endogenous oxytocin system on heroin choice. Thus, drugs were administered in the following order: 1 mg/kg oxytocin, vehicle, 3 mg/kg oxytocin, vehicle, 5 mg/kg L-368,899. Orexin rats received vehicle or DORA-12 (10 or 30 mg/kg, i.p.). Two vehicle tests were conducted to account for the different volumes required to deliver the low (1 ml/kg) versus high (3 ml/kg) dose of the DORA compound due to solubility limits. Drugs were administered in the following order: 1 ml/kg vehicle or 10 mg/kg DORA, followed by 3 ml/kg vehicle or 30 mg/kg DORA. Since no statistical differences between the low- and high-volume vehicle tests were observed, vehicle test data were averaged prior to statistical analyses.

### Cued reward seeking test

After completing choice testing, rats were returned to FR3 self-administration procedures identical to those during training. During the first 30 min of each session, the food lever was available, followed 10 min of levers retracted, and then the heroin lever was available for 2 h. After at least three reacquisition sessions, rats underwent a single cued reward seeking test. During the test, both food and heroin levers were simultaneously available for 1 h. Pressing on each lever resulted in cue delivery (food-tone or heroin-light) on an FR3 schedule (with lever retraction during cue delivery, as during earlier self-administration sessions), but no rewards. Note that repeated testing is not feasible under these conditions, as responding extinguishes rapidly; hence, a single dose of each agent was selected for testing. Each rat received either vehicle (water or 5% DMSO + 40% captisol in water), 1 mg/kg oxytocin, or 30 mg/kg DORA-12, 30 min prior to the cue test. The 1 mg/kg dose of oxytocin has been commonly used to reduce cued reward seeking in other studies [17, 18]. The 30 mg/kg dose of DORA-12 was chosen because there is no precedent in the literature for the efficacy of this compound on drug seeking, but studies using other orexin receptor antagonists with structural similarity to DORA-12 (e.g., suvorexant) indicate that a dose of 30 mg/kg may be more effective than 10 mg/kg at reducing drug seeking [48, 63].

### Immunohistochemistry and cell counts

The number of orexin neurons and oxytocin neurons were counted in the brains of male rats from our previous study [6], which underwent similar behavioral procedures, but were never exposed to pharmacological agents targeting the orexin or oxytocin systems. These rats were transcardially perfused with phosphate buffered saline (PBS) followed by formalin. Brains were post-fixed for 1 h, and cryoprotected in 30% sucrose prior to sectioning. Brain slices (40 µm) were incubated in blocking buffer consisting of PBS-Triton X-100 (0.3%; PBS-T) with 2% normal donkey serum for 2 h at room temperature, and incubated at 4 °C overnight with mouse anti-oxytocin (1:1000, Millipore Cat# MAB5296, RRID:AB_2157626) or mouse anti-orexin A (1:500, Santa Cruz Biotechnology Cat# sc-80263, RRID:AB_1126868). After 3 PBS washes, sections were incubated for 2 h with donkey anti-mouse Alexafluor^®^ 594 (1:500; Jackson ImmunoResearch Labs Cat# 715-585-151, RRID:AB_2340855) secondary antibody. Sections were mounted onto slides and coverslipped with ProLong Diamond Antifade mounting medium with DAPI (Invitrogen Cat# P36971). Images were acquired with a slide scanner microscope (Olympus VS120; 10x air objective) and imported into Imaris software (Imaris, RRID:SCR_007370). Oxytocin neurons were counted semi-automatically using the Imaris spot detection function. The number of oxytocin cells in the PVN was counted in both hemispheres and averaged across two sections per animal. For orexin counts, images were acquired using a Zeiss Axio Zoom V16 microscope. Tiled photographs were compiled at ×20 magnification using Zen 2 imaging software (Carl Zeiss Microscopy). As in our previous studies [60, 64], the number of orexin cells was counted separately in the LH versus the dorsomedial hypothalamus (DMH) and perifornical area (PF) orexin cell fields across both hemispheres using the manual quantification functionality of Zen 2 software, and averaged across three sections per animal. Orexin subfields were delineated as described previously [60, 64] by drawing a line 100 microns lateral to the fornix, with the field medial to this line representing PF/DMH and lateral to this line representing the LH orexin field. Counts of oxytocin and orexin cells were conducted by separate investigators blind to the experimental condition of the animals.

### Statistical analyses

All statistical analyses were performed in Prism (GraphPad Prism, RRID:SCR_002798; V9.1). Differences in behavioral economics variables were assessed using 2-way repeated measures (RM) analyses of variance (ANOVA) with reward (food and heroin) as the within-subjects factor and sex as the between-subject factor. Choice data and lever presses for food or heroin during choice testing were analyzed using a one-way RM-ANOVA with treatment as within-subject factor or a 2-tailed paired *t* test where appropriate. Cued reward seeking test data were analyzed using a 2-way RM-ANOVA with lever (heroin or food) as within-subject factor and treatment as between-subject factor, followed by Sidak post hoc tests. Linear relationships between variables were analyzed using Pearson’s Correlation coefficient.

## Results

### Motivation for heroin exceeds that for food

**Fig. 2:**
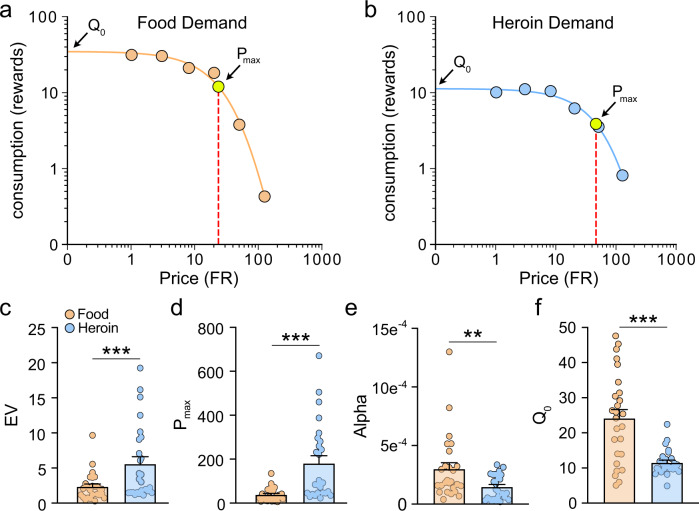
Heroin is valued more highly than food, assessed using behavioral economics principles. Representative demand curves for food (**a**) and heroin (**b**) demonstrate more inelastic demand for heroin compared to food in rats. **c** The essential value (EV) and **d** maximum price rats were willing to pay to defend desired consumption (*P*_max_) were higher for heroin than for food. **e** Demand elasticity (*α*) and **f** consumption at null cost (*Q*_0_) were higher for food than for heroin. **p < 0.01, ***p < 0.001 comparing food versus heroin (2-tailed paired *t* test). Data are shown as mean ± SEM (**c**–**f**).

Representative demand curves for food and heroin self-administration over increasing price points from a single animal are shown in Fig. 2a, b. Responding during self-administration and behavioral economics phase are shown in Supplementary Fig. 1a, b. Consistent with our previous work [6], rats were more motivated for heroin than for food, by several measures of behavioral economics variables. EV (Fig. 2c; 2-way RM-ANOVA: main effect of reward, *F*_(1,24)_ = 15.39, *p* = 0.0006; paired *t* test: *t*_(25)_ = 3.826, *p* = 0.0008) and *P*_max_ (Fig. 2d; 2-way RM-ANOVA: main effect of reward, *F*_(1,24)_ = 20.77, p = 0.0001; paired *t* test: *t*_(25)_ = 4.582, *p* = 0.0001) were significantly higher for heroin than for food, and heroin α was lower than food α (Fig. 2e; 2-way RM-ANOVA: main effect of reward, *F*_(1,24)_ = 12.30, *p* = 0.0018; paired *t* test: *t*_(25)_ = 3.14, *p* = 0.0043). These results indicate that rats had a more inelastic demand curve for heroin, suggesting that under these experimental conditions, rats treat heroin as a necessity and food as a luxury (see Fig. 1a, b for explanation of concepts and variables). Food *Q*_0_ was higher than heroin *Q*_0_ (Fig. 2f; 2-way RM-ANOVA: main effect of reward, *F*_(1,24)_ = 22.63, *p* < 0.0001; paired *t* test: *t*_(25)_ = 4.8436, *p* < 0.0001), indicating that rats respond significantly more for food than for heroin under “free” access conditions, consistent with our prior work [6].

As expected based on our prior work, certain behavioral economics variables correlated with others for a given reinforcer (heroin vs. food; Supplementary Fig. 2). In addition, some correlations in behavioral economics variables between reinforcers were observed; however, no behavioral economics variables correlated with choice (Supplementary Fig. 2). The latter is consistent with our prior conclusion that choice is a distinct measure of addictive behavior, possibly reflecting more cognitive, as opposed to motivational, aspects of addictive behavior [6]. Further analyses revealed that heroin demand was less elastic than food demand in female rats (Supplementary Fig. 3c; 2-way RM-ANOVA: reward × sex interaction, *F*_(1,24)_ = 7.18, *p* = 0.0131; *α* food versus *α* heroin: *p* = 0.0004). However, no significant differences between sexes were observed in any behavioral economics variables (Supplementary Fig. 3).

Heroin preferring rats were defined as those showing >65% heroin choice, and food preferring rats were defined by <35% heroin choice, leaving those choosing 35–65% as equal preferers. Seven of the ten heroin preferring rats in this study were female; equal preferring rats were roughly equivalent in sex (5 females, 4 males), whereas pellet preferring rats were predominantly male (6 of 7 total). Thus, in this study, ~38% of the population was heroin preferring, ~35% equal preferring, and ~27% pellet preferring, yielding a population average of 56 ± 6% heroin choice (Supplementary Fig. 1c). Furthermore, females had a significantly higher heroin choice baseline than males prior to commencement of choice testing (Supplementary Fig. 3e; unpaired *t* test: *t*_(24)_ = 2.21, *p* = 0.0366).

### Neither oxytocin nor orexin mediate opioid choice

**Fig. 3:**
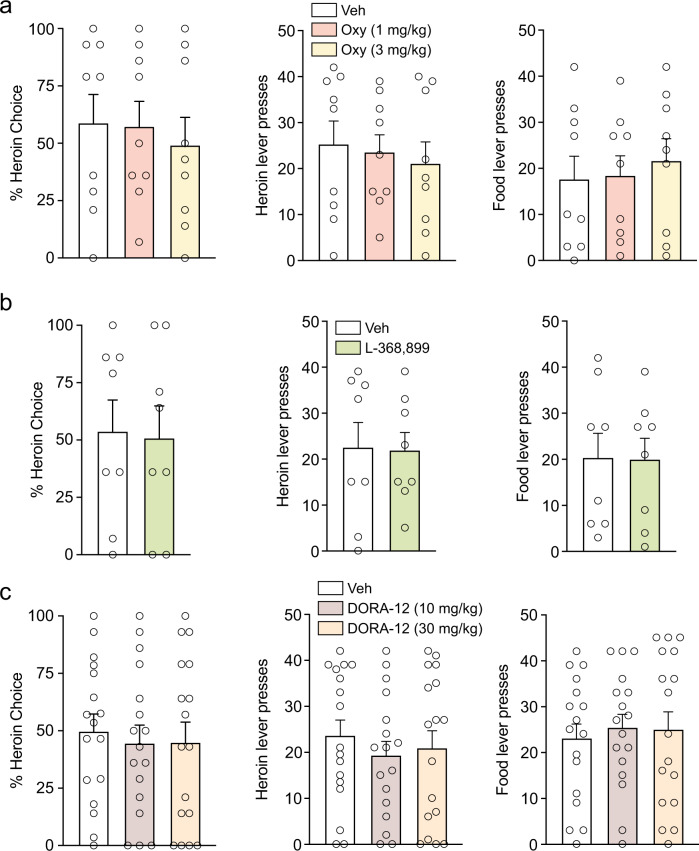
Oxytocin and orexin agents do not alter heroin choice. Neither the low dose (1 mg/kg) or the high dose (3 mg/kg) of oxytocin altered heroin choice (**a**, left), or lever presses for heroin (**a**, middle) or food (**a**, right). The oxytocin antagonist, L-368,899 (5 mg/kg), did not alter choice (**b**, left), lever presses for heroin (**b**, middle) or food (**b**, right). The dual orexin receptor antagonist DORA-12 did not alter heroin choice (**c**, left), lever presses for heroin (**c**, middle) or food (**c**, right), at either the low (10 mg/kg) or high dose (30 mg/kg). Data shown as mean ± SEM.

After choice behavior stabilized and preference phenotypes were defined for individual rats, choice testing commenced. Different pharmacological agents were used to assess the involvement of the hypothalamic neuropeptide systems, oxytocin and orexin, in opioid choice. Oxytocin (1 or 3 mg/kg, i.p.) or vehicle was administered 30 min prior to the choice test session in rats assigned to the oxytocin study. A within-subjects design was used, with at least two drug-free choice sessions in between tests. No treatment effects of oxytocin were observed on opioid choice (Fig. 3a, left), heroin lever presses (Fig. 3a, middle) or food lever presses (Fig. 3a, right) under these conditions. These same rats then underwent an additional test with the oxytocin antagonist L-368,899 (5 mg/kg, i.p.) or vehicle, and there were no effects on opioid choice (Fig. 3b, left), heroin lever presses (Fig. 3b, middle) or food lever presses (Fig. 3b, right).

In rats assigned to the orexin study, the dual orexin receptor antagonist DORA-12 (10 or 30 mg/kg, i.p.) or vehicle was administered 30 min prior to the choice test session, again using a within-subjects design, with drug-free choice sessions separating each test. There were no effects of DORA-12 on opioid choice (Fig. 3c, left), heroin lever presses (Fig. 3c, middle) or food lever presses (Fig. 3c, right) under these conditions.

### Oxytocin and orexin systems bidirectionally regulate opioid seeking in the presence of competing cues

**Fig. 4:**
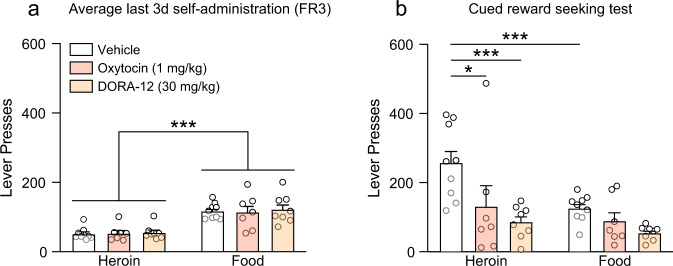
Oxytocin and DORA-12 reduce drug seeking triggered by heroin cues. Responding did not differ between treatment groups during the FR3 sessions preceding the cued reward seeking test (**a**). Under conditions where both heroin and food cues are available, rats seek heroin at higher rates than food (**b**). Oxytocin (1 mg/kg) and DORA-12 (30 mg/kg) reduced heroin seeking, but not food seeking, in the presence of both heroin and food cues. *p < 0.05, ***p < 0.001 versus heroin-vehicle group (Two-way RM ANOVA followed by Sidak post hoc test). Data are shown as mean ± SEM.

After choice testing was complete, all rats were returned to food and heroin self-administration (on FR3) for at least 3 sessions before the cued reward seeking test. During this final test, rats were able to respond on both heroin and food levers, which delivered the heroin- or food-related cues (on FR3) but did not deliver the rewards. Thus, this was a cue extinction test, similar to other forms of cued reinstatement [6, 65], conducted during acute (~24 h) opioid withdrawal under conditions of competing reward-related cues. Oxytocin (1 mg/kg), the orexin antagonist DORA-12 (30 mg/kg), or vehicle were injected 30 min prior to the test. Figure 4a shows the average of active lever presses during the last 3d of self-administration preceding the cued reward seeking test. Lever pressing for food was higher than heroin (2-way ANOVA; Lever: *F*_(1,21)_ = 49.93, *p* < 0.0001), but there were no a priori differences between treatment groups.

During the cued reward seeking test, rats responded at higher rates on the heroin lever for the heroin cue (Fig. 4b; 2-way RM-ANOVA: main effect of lever, *F*_(1,21)_ = 16.15, *p* = 0.0006) compared to the food lever, consistent with the higher motivation to seek heroin when both cues were present. Treatment with either oxytocin or orexin agents produced a significant reduction in responding (2-way RM ANOVA: main effect of drug, *F*_(2,21)_ = 5.662, *p* = 0.0108; lever × drug interaction, *F*_(2,21)_ = 3.646, *p* = 0.0437), and post-hoc analyses indicated that this reduction in responding was specific to the heroin lever (oxytocin: *p* = 0.0183, DORA-12: *p* = 0.0006, compared to Veh group) with no significant effects on the food lever. Furthermore, responding for the heroin cue was greater than responding for the food cue only in the Veh group (*p* = 0.0003). These results indicate that activating the oxytocin system with exogenous oxytocin, or inhibiting the orexin system with DORA-12, is capable of normalizing rates of reward seeking in the presence of heroin cues to those of food.

### Oxytocin and orexin cell numbers differentially associate with food and heroin demand

We counted the number of orexin neurons within the LH and DMH/PF and the number of oxytocin neurons within the PVN in the brains of rats from our previous study [6]. Importantly, these rats were never exposed to pharmacological agents targeting the orexin or oxytocin systems, which might have impacted expression levels of these neuropeptides [66, 67]. Consistent with the lack of effect of oxytocin and DORA-12 on opioid choice, we observed no correlation between the numbers of oxytocin or orexin neurons and choice behavior (Supplementary Fig. 4a, b).

**Fig. 5:**
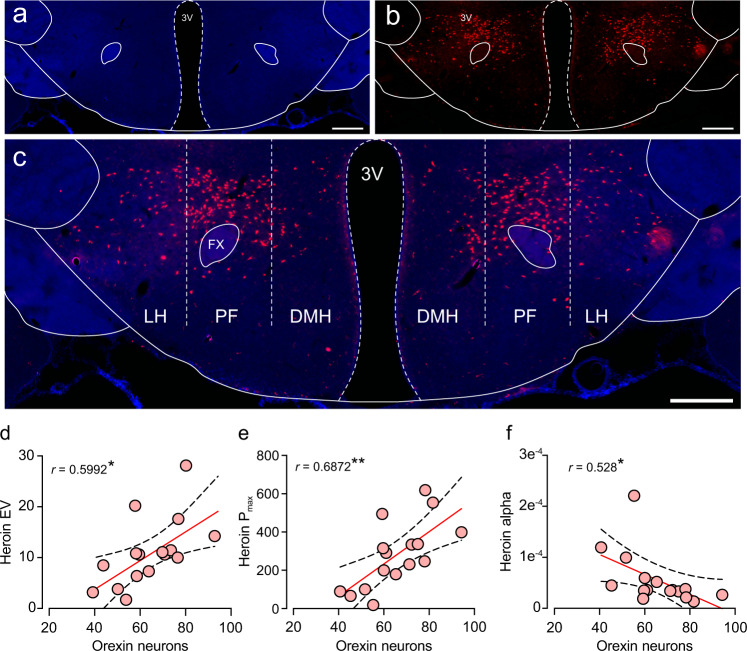
The number of orexin neurons in the lateral hypothalamus correlates with behavioral economics indicators of heroin motivation. **a** Representative images of the neuronal stain (DAPI), **b** orexin immunofluorescence, and **c** the overlay are shown, depicting the anatomical boundaries between lateral hypothalamus (LH) versus the dorsomedial (DMH) and perifornical (PF) regions (3V third ventricle, FX fornix. Scale bar = 500 µm). The number of LH orexin neurons positively correlated with heroin EV (**d**) and *P*_max_ (**e**), and negatively correlated with heroin *α* (**f**). This profile implicates the LH orexin system as a driver of the heroin motivational state. *p < 0.05, **p < 0.01 significant correlation.

**Fig. 6:**
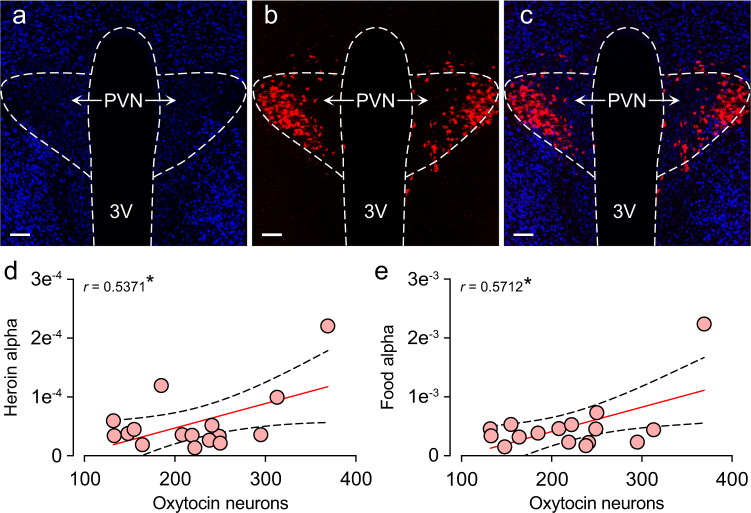
The number of oxytocin neurons in the paraventricular hypothalamus correlates with both heroin and food demand elasticity. **a** Representative images of the hypothalamus (DAPI), **b** oxytocin immunofluorescence, and **c** the overlay showing the anatomical boundaries for the paraventricular hypothalamus (PVN). 3V third ventricle. Scale bar = 100 µm. The number of PVN oxytocin neurons positively correlated with both heroin *α* (**d**) and food *α* (**e**). This profile implicates the oxytocin system as a limiter of motivational states. *p < 0.05 significant correlation.

We then examined correlations between these counts and the behavioral economics variables from each individual rat’s demand curves for heroin and food, as well as reward seeking during the cue test. Figure 5a–c shows a representative image of the hypothalamic orexin field. We found that the number of LH orexin neurons positively correlated with heroin EV (Fig. 5d; Pearson’s *r* = 0.5992, *p* = 0.0142) and *P*_max_ (Fig. 5e; Pearson’s *r* = 0.6827, *p* = 0.0036), and negatively correlated with heroin *α* (Fig. 5f; Pearson’s *r* = -0.528, *p* = 0.0355). Interestingly, no food variables correlated with orexin cell counts (data not shown). Moreover, the number of orexin neurons in the DMH/PF did not correlate with any behavioral economics variables for heroin or food (Supplementary Fig. 4c–e). This underscores the specificity of the LH orexin system in motivation for heroin versus natural reward (food). We then examined the correlation between oxytocin cell numbers and the behavioral economics variables. Figure 6a–c shows representative images of oxytocin neurons in the PVN. Oxytocin cell counts positively correlated with both heroin *α* (Fig. 6d; Pearson’s *r* = 0.5371, *p* = 0.0312) and food *α* (Fig. 6e; Pearson’s *r* = 0.5712, *p* = 0.0209). No other variables correlated with oxytocin cell counts (data not shown). This suggests a possible generalized involvement of the oxytocin system in motivation for both drug and natural rewards.

## Discussion

Altogether these results provide evidence that two hypothalamic neuropeptides, oxytocin and orexin, bidirectionally regulate the ability of opioid cues to bias choice in acute (~24 h) withdrawal. We confirmed previous findings in a similar choice model that rats are willing to work more for heroin than natural reward (food) [6]. Indeed, demand for heroin was more inelastic than demand for food, suggesting that the rats in this study treat heroin as a necessity and food as a luxury item. Multiple variables derived from individual demand curves pointed to increased motivation for heroin relative to food. The essential value (EV) of heroin was higher than that of food, with EV reflecting a normalized *α*, allowing us to directly compare the elasticity of demand between these two different rewards. *P*_max_, or the maximum price the animal is willing to pay (in lever presses) to defend desired consumption (*Q*_0_), was also higher for heroin than food. Moreover, heroin *α* correlated with both the number of orexin and oxytocin neurons, indicating that these hypothalamic systems underlie motivation for heroin.

A few preclinical studies have investigated the relationship of behavioral economics variables and choice between drugs and non-drug alternative rewards. Early work suggested that rodents value sweet rewards (e.g., sucrose or saccharin) as much or more than drug rewards [68, 69]. In this study, the type of home cage chow was similar in composition to the type of food pellets available in the operant chambers, and rats were not food-deprived. This may account, in part, for the more equal preference between food and heroin observed under the choice conditions used here. Alternatively, more recent work suggests that the interval between choice trials, and the pharmacodynamics of the drug (in this case, heroin), are critical factors that impact choice behavior [70, 71]. When comparing between two different rewards like food and heroin, EV is a useful metric, as it provides a normalization of the primary variable α, indicative of demand elasticity. In this domain, previous studies have demonstrated positive correlations between cocaine or heroin EV and drug choice, and negative correlations between food or saccharin EV and drug choice [59, 62]. In the present study, heroin choice did not correlate with any behavioral economics variables, indicating that these are fundamentally separate metrics of OUD severity. Furthermore, heroin EV was higher than food EV, whereas previous studies have found food or saccharin EV to be higher than drug (cocaine or heroin) EV [59, 62]. Consistent with our prior work [6], food *α* was higher than heroin *α*, again pointing to a higher motivational state for heroin than food, contrary to other reports [59, 72, 73]. Nonetheless, this underscores the enhanced face validity of this behavioral economics choice model for OUD.

The hypothalamus, especially its oxytocin and orexin neuropeptide systems, has been strongly implicated in addiction-related behaviors [31, 41, 74, 75]. Systemic administration of the Ox1R antagonist SB-334867 reduces opioid taking during heroin self-administration, opioid breakpoint under progressive ratio tests, and cued opioid seeking [76, 77]. Systemic administration of SB-334867 also decreases behavioral economic measures of motivation for fentanyl and remifentanil [39, 40, 76]. Despite the increasing body of literature on the efficacy of the orexin-based therapies targeting Ox1R [38], less is known on the efficacy of dual orexin receptor antagonism on drug taking and seeking. Emerging data in this area is promising; for instance, acute treatment with the DORA almorexant attenuates cocaine- and methamphetamine-induced conditioned place preference [50]. Similarly, acute treatment with the DORA suvorexant reduces cocaine taking during self-administration, hedonic responses to cocaine, cocaine-induced dopamine elevations, as well as cocaine-induced impulsive behavior [47, 48]. Our results similarly indicate that acute treatment with DORA-12 reduces reward seeking in the presence of heroin cues, but not simultaneously available food cues, underscoring the apparent specificity of the orexin system in facilitating motivated behavior for highly salient rewards, including drugs of abuse [78]. Our data are also consistent with the finding that acute treatment with an Ox2R antagonist specifically reduces heroin taking during self-administration, but not sucrose taking [42]. Collectively, these findings suggest that DORA-based therapies harbor great potential for treating substance use disorders (SUDs). Importantly, there are currently three DORAs being used clinically for the treatment of insomnia (e.g., suvorexant, lemborexant, and daridorexant) that could easily be repurposed as anti-SUD therapeutics.

Both clinical and preclinical studies support the therapeutic potential of oxytocin-based medications for SUDs, including OUD [79]. A recent clinical trial showed that a single intranasal oxytocin dose reduced craving and withdrawal scores in heroin users during abstinence [27]. Early preclinical studies showed that systemic administration of exogenous oxytocin reduces both the acquisition and maintenance of heroin self-administration in rats [80, 81] and decreases naloxone-precipitated withdrawal symptoms in morphine-dependent mice [82]. Moreover, intracerebroventricular administration of oxytocin reduces reinstatement of oxycodone conditioned place preference [83, 84], and systemic administration of Carbetocin, an oxytocin analog, reduces stress- [85] and prime-induced [86] reinstatement of morphine conditioned place preference. Strikingly, to date, no preclinical studies have investigated the efficacy of systemic oxytocin administration on heroin seeking. Our results show that acute oxytocin treatment reduces opioid seeking in the presence of opioid cues competing with cues associated with natural rewards, expanding our knowledge of the domains by which oxytocin exerts multimodal anti-addictive properties [87].

Interestingly, neither oxytocin nor DORA altered choice behavior when heroin and food were present. Consistent with these negative behavioral findings, neither oxytocin nor orexin cell numbers correlated with choice behavior. However, both compounds were effective at reducing opioid seeking in the presence of heroin cues (food cues simultaneously available). Thus, activating the oxytocin system or inhibiting the orexin system reduces reward seeking triggered by heroin cues to levels similar to those of food cues. A couple of key differences between the test conditions may account for these apparent disparate results. First, during the choice tests, response rates are low and rewards are available and being consumed. This type of behavior may be less amenable to disruption than behavior under conditions like the cue test, where competing heroin and food cues are available, but not their respective rewards, and response rates are high. Furthermore, the particular choice conditions used here do not capture all elements of a free economy, as heroin was only available during operant sessions [88–90]. This may have constrained choice behavior, and enhanced its resistance to disruption by pharmacological agents. Nonetheless, our choice model provides valuable insight into the ability of oxytocin and orexin agents to alter responding for heroin and food when both rewards are available. Choice models provide benefits over single operant self-administration procedures because of their inherent sensitivity to detect general changes in motor performance and motivation [91, 92].

Despite the considerations noted above, the specificity of the effects of DORA-12 and oxytocin on cued heroin seeking suggest that these therapies may be particularly effective at preventing relapse triggered by drug-associated cues. Though we did not explore the neural circuitry underlying these therapeutic effects here, the fact that DORA-12 and oxytocin affected the same behavioral outcomes, but in opposite directions, raises the possibility that their actions are mediated via similar brain regions and mechanisms. A potential common site of action is the basolateral amygdala; orexin inputs to the basolateral amygdala, particularly involving the Ox1R, have been implicated in cued reinstatement of drug seeking [93], and evidence suggests they also process stress-related information within this circuit [94, 95], consistent with a role for orexin in stress-induced forms of relapse [96, 97]. Intranasal oxytocin therapy for the treatment of posttraumatic stress disorder has also been shown to normalize functional connectivity in basolateral amygdala circuits [98]. Moreover, binding at both orexin and oxytocin receptors engages downstream phospholipase C (PLC) pathways [99, 100], and dysfunction of the PLCβ1 isoform is implicated in stress and addiction [94, 101]. While there are many other brain regions and intracellular pathways that may be part of the circuitry mediating the observed therapeutic effects of oxytocin and DORA-12 on cued heroin relapse [15, 84], more work is needed to identify not only the circuit-level mechanisms, but also the molecular and G-protein signaling mechanisms underlying these results.

Although neither oxytocin nor orexin cell counts correlated with heroin seeking during the cued reward seeking test, correlations with α were observed. The variable α has been shown repeatedly to correlate with drug-seeking for multiple drugs of abuse [61] and is associated with the propensity to develop a more severe SUD phenotype in cocaine self-administering rats [102]. Consistent with several previous studies indicating a functional dichotomy for LH versus DMH/PF orexin cells in reward and stress processes, respectively [60, 64, 103, 104], we found that the number of LH (but not DMH/PF) orexin cells negatively correlated with heroin α. Interestingly, we observed no relationship between orexin cell numbers in either LH or DMH/PF and food α, indicating an apparent specific relationship between the LH orexin system in motivation for heroin. Moreover, positive correlations were observed between LH (but not DMH/PF) orexin cell numbers and heroin *P*_max_. Notably, the maximum amount of effort exerted to maintain drug consumption at *P*_max_, has received considerable attention as a reliable predictor of SUD severity in humans, particular for alcohol. In humans with recent binge drinking episodes, the demand curve for alcohol becomes more inelastic [105], and even without binging, behavioral economic metrics correlated with weekly alcohol intake [106], again supporting the face validity of behavioral economics to assess SUD severity in humans [107]. Collectively, these findings implicate the orexin system as a potential biomarker of propensity to develop SUD.

In contrast with the negative correlation observed between orexin cell numbers and heroin α, oxytocin cell numbers positively correlated with both heroin α and food α, highlighting the bidirectional functional role of these systems in motivation and suggesting a more general involvement of the oxytocin system in motivation for both drug and non-drug rewards. For instance, acute administration of oxytocin decreases sucrose taking during self-administration as well as seeking triggered by sucrose cues [57]. While this conflicts with our present finding that oxytocin specifically reduces relapse for heroin, but not food, cues, it is consistent with our finding that oxytocin counts correlated with both heroin and food α.

Finally, both oxytocin and orexin play an important role in arousal and sleep, and soporific effects of exogenous oxytocin and DORAs have been reported under some circumstances. However, it is unlikely that the behavioral effects of oxytocin and DORA-12 on opioid-seeking behavior observed here are the result of impairment in general activity, as (1) these compounds had no effect on choice behavior, a task that required sustained operant responding across the session; and (2) in the case of DORA-12, several studies have reported no effect of similar compounds on animals’ locomotor activity profile, low-effort drug intake, or performance on cognitive tasks that require a high level of task engagement and responding [47–49]. Nevertheless, any development of pharmacological strategies that target the oxytocin and/or orexin systems will need to be mindful of potential unwanted effects on general arousal and related outcomes [44].

In sum, these results point towards the hypothalamic oxytocin and orexin systems as two potential therapeutic targets capable of bidirectionally regulating reward seeking in a world of competing drug and non-drug cues. Since relapse rarely occurs in an isolated setting where only drug cues are available, these results have implications for relapse that occurs under conditions where alternatives are available. Future studies should thoroughly examine the potential to exploit the oxytocin and orexin systems to achieve remission in SUDs, by biasing choice towards natural rewards and reducing craving triggered by drug-associated cues.

## Supplementary information

Supplemental material
