# Supplementary material for: Oxytocin and orexin systems bidirectionally regulate the ability of opioid cues to bias reward seeking
Source: Transl Psychiatry. 2022 Oct 4;12:432. doi: 10.1038/s41398-022-02161-z (PMC9532415; doi:10.1038/s41398-022-02161-z)
Supplement: Supplementary file 1 — Supplemental material [file 41398_2022_2161_MOESM1_ESM.pdf]

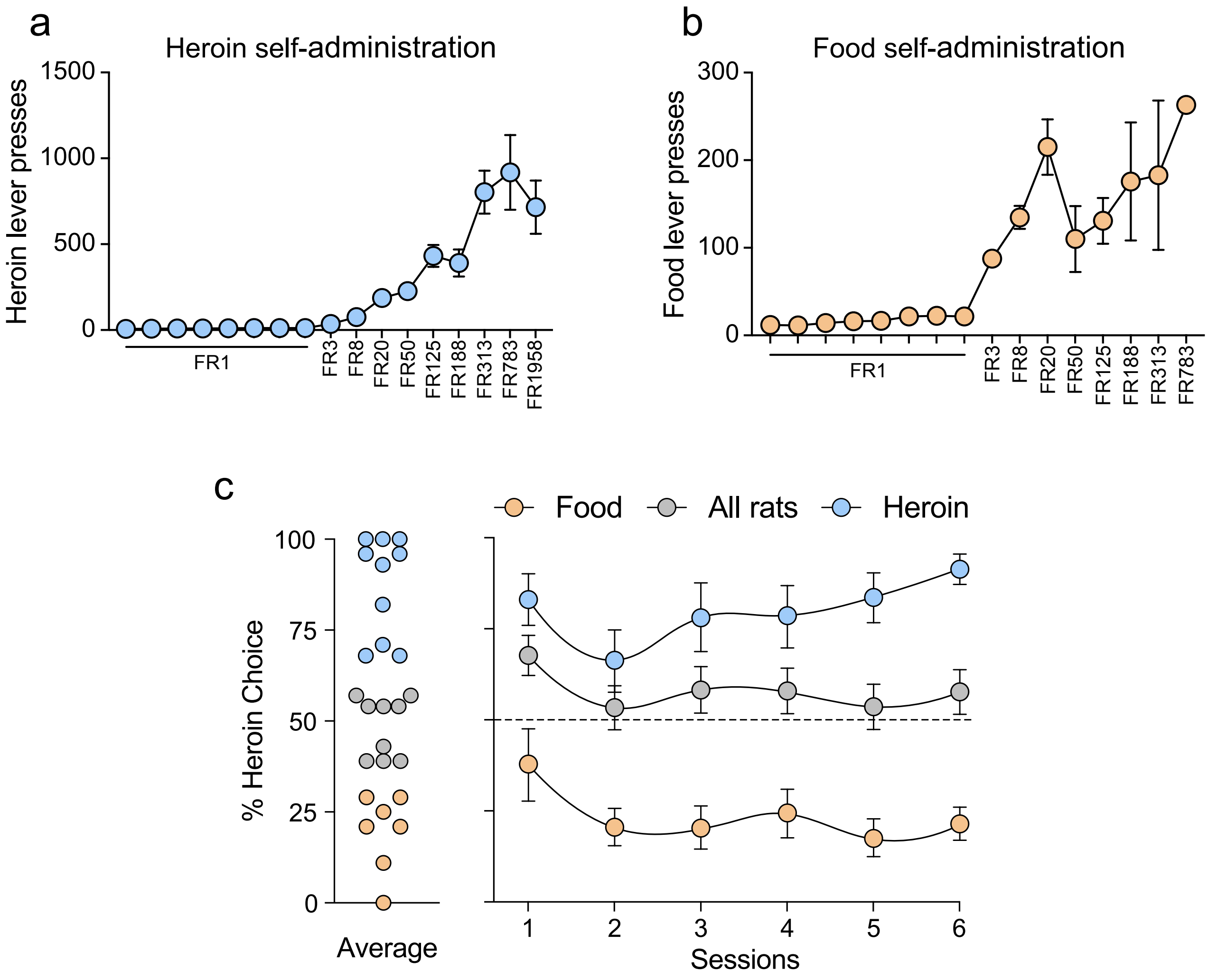

Supplementary Figure 1. Responding during self-administration, behavioral economics, and choice procedures. Acquisition of heroin (a) and food (b) self-administration occurred on an FR1 and continued over subsequent FR price points for behavioral economics analyses. Responding during the choice phase (c) revealed that rats choose heroin versus food ~50% of the time, with individual subpopulations preferring heroin vs. food.

p-value: | *

r-value: | *

		Heroin EV	Heroin P_{max}	Heroin α	Heroin Q_0	Choice	Food EV	Food P_{max}	Food α	Food Q_0
Heroin	EV		$9.5e^{-17}$	$3.5e^{-7}$	0.34	0.05	$5.1e^{-4}$	$5.4e^{-4}$	0.06	0.76
Heroin	P_{max}	0.97		$3.9e^{-7}$	0.08	0.09	$1.8e^{-4}$	$1e^{-3}$	0.06	0.92
Heroin	α	-0.82	-0.82		0.24	0.28	0.02	0.02	0.02	0.57
Heroin	Q_0	-0.2	-0.35	0.24		0.18	0.97	0.73	0.31	0.83
	Choice	0.38	0.34	-0.22	-0.27		0.84	0.89	0.52	0.68
Food	EV	0.63	0.67	-0.45	$9e^{-3}$	-0.04		$8.9e^{-7}$	$1e^{-3}$	0.59
Food	P_{max}	0.63	0.61	-0.47	0.07	-0.03	0.80		0.02	0.05
Food	α	-0.38	-0.37	0.45	-0.21	0.13	-0.61	-0.47		0.09
Food	Q_0	-0.06	-0.02	0.12	-0.04	0.09	0.11	-0.39	-0.34	

Supplementary Figure 2. Correlation matrix comparing all behavioral variables. Behavioral economic variables correlate with each other. By contrast, choice does not correlate with any of the behavioral economic variables. n = 26 rats. * $p < 0.05$ significant correlation (Pearson's r) compared to the null hypothesis of no relationship between variables.

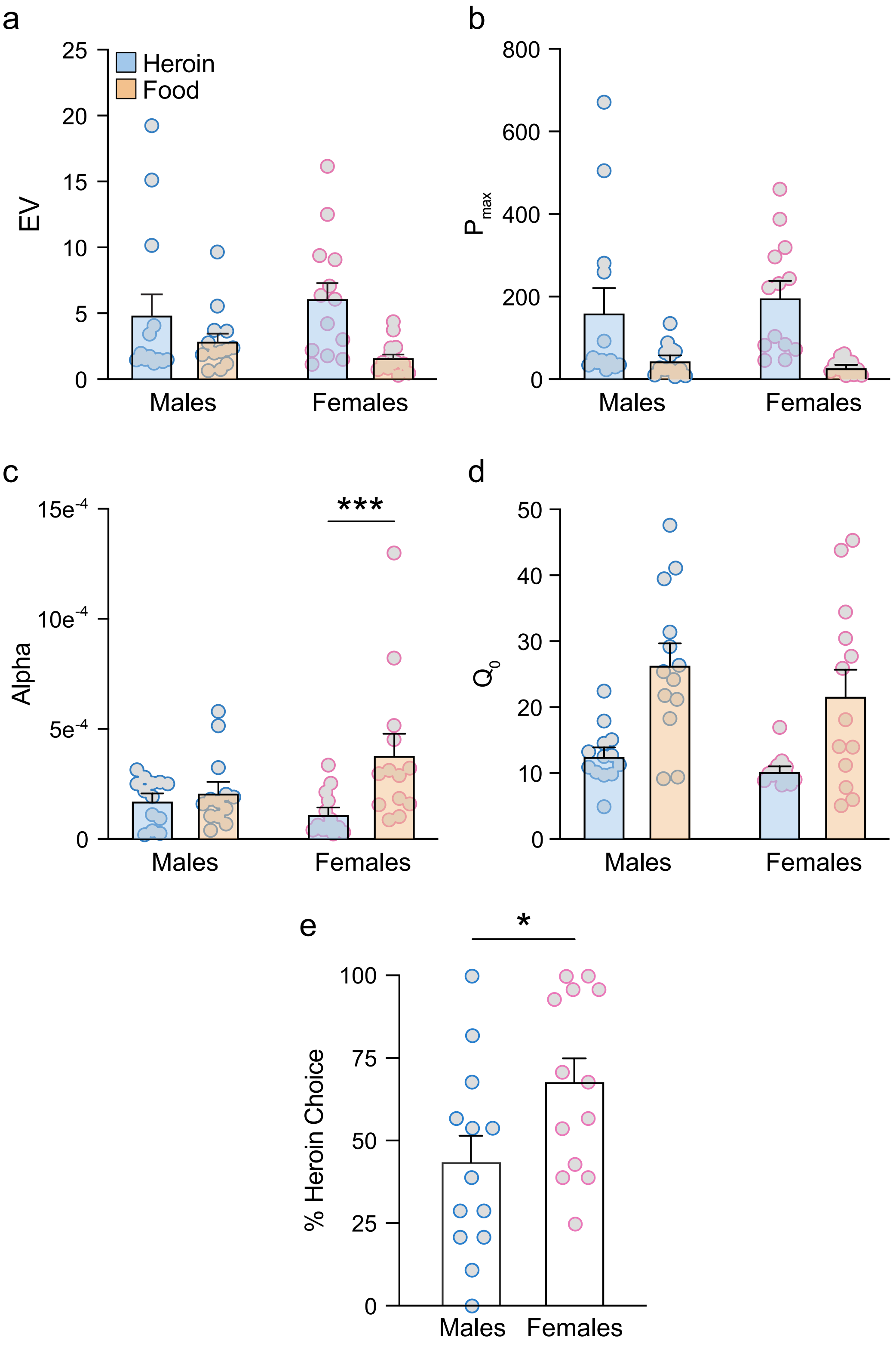

Supplementary Figure 3. Behavioral economics variables and choice behavior disaggregated by sex. The essential value (a) and Pmax (b) of food or heroin is not different between male and female rats. (c) Female rats have significantly less elastic demand for heroin than food. (d) Male and female rats do not differ in their consumption of food or heroin at no cost (Q_0). (e) Females choose heroin more than males. n = 26 rats. *** $p < 0.001$ comparing heroin and food variables. * $p < 0.05$ comparing males and females. Data are presented as mean ± SEM.

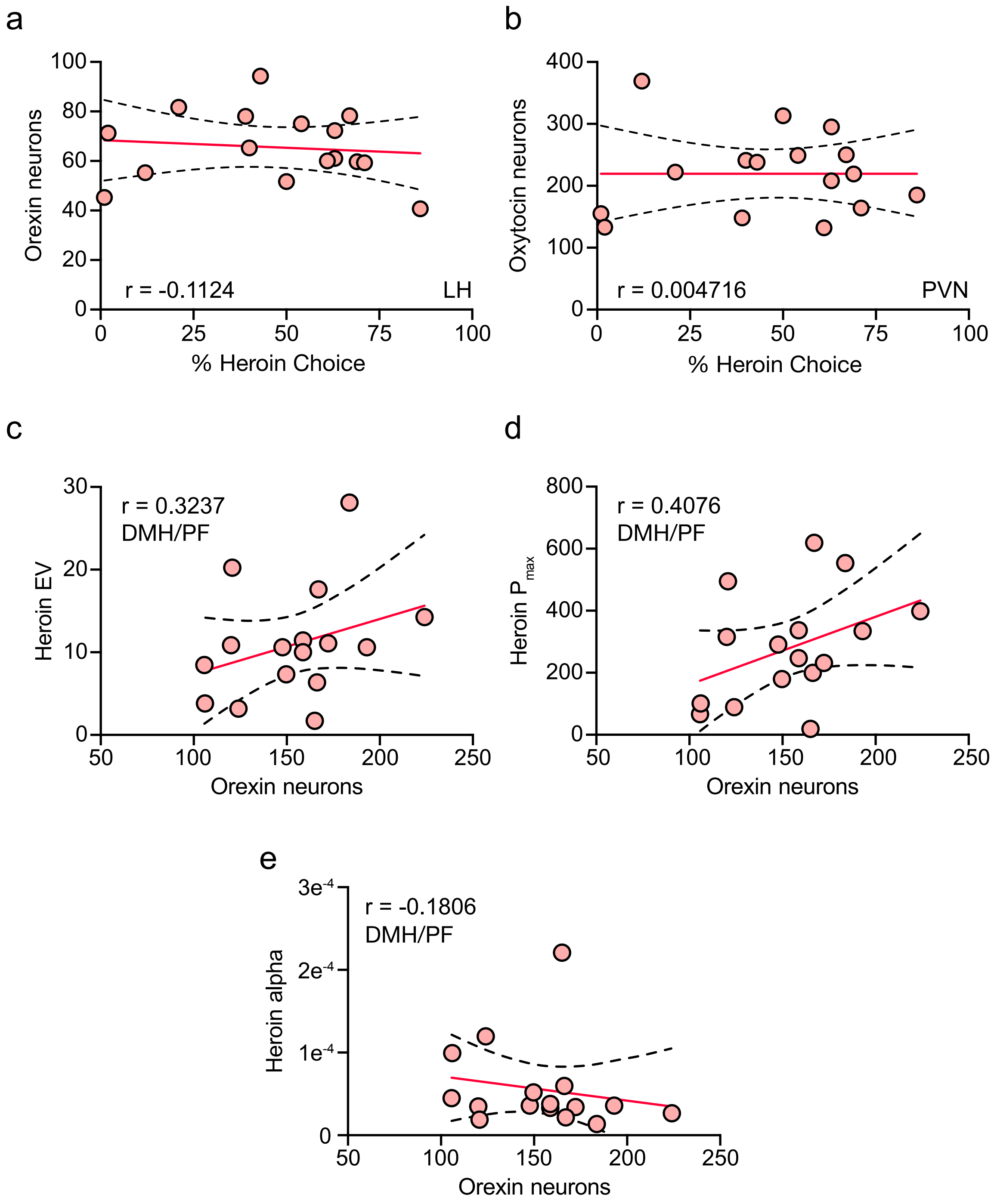

Supplementary Figure 4. Additional correlational analyses for orexin and oxytocin neurons. The number of orexin neurons in the lateral hypothalamus (LH) does not correlate with heroin choice (a); neither does the number of oxytocin neurons in the paraventricular nucleus (PVN) of the hypothalamus (b). The number of orexin neurons in the dorsomedial (DMH) and perifornical (PF) hypothalamic regions does not correlate with the essential value of heroin (c), heroin Pmax (d) or heroin alpha (e).
